# 

**Published:** 1864-01

**Authors:** 


Volume XXI. Number 1.

### THE CHICAGO

### MEDICAL JOURNAL.

DE LASKIE MILLER, M. D.,
EPHRAIM INGALS, M. D.,
Editors and Proprietors.

JANUARY, 1864.

CHICAGO:
J. C. W. BAILEY, BOOK AND JOB PRINTER, 130 CLARK STREET.
1864.

